# Supplementary material for: A feedback loop governs the relationship between lipid metabolism and longevity
Source: eLife. 2020 Oct 20;9:e58815. doi: 10.7554/eLife.58815 (PMC7575325; doi:10.7554/eLife.58815)
Supplement: Supplementary file 1. — * indicates the 95% CI for the ratio of median survival. [file elife-58815-supp1.docx]

**Supplemental File 1**. **List of all p-values and 95% Confidence Interval (CI) of the difference between means for all Figures.**

| **Figure** | **Group Comparison** | **p-value** | **95% CI of difference between means** |
| --- | --- | --- | --- |
| 1C | vehicle vs 5-HT | 0.013 | 11.4 to 91.5 |
| 1E | wild-type vector RNAi vs *flp-7^tg^* vector RNAi | p<0.001 | -80.1 to -60.0 |
| 1E | wild-type *atgl-1* RNAi vs *flp-7^tg^ atgl-1* RNAi | 0.0675 | -41.5 to 1.0 |
| 1G | vehicle vector RNAi vs 5-HT vector RNAi | p<0.001 | -75.5 to -55.2 |
| 1G | vehicle *atgl-1* RNAi vs 5-HT *atgl-1* RNAi | 0.9322 | -7.9 to 12.5 |
| 1H | wild-type vector RNAi vs *flp-7^tg^* vector RNAi | 0.002 | 1.9 to 6.2 |
| 1H | wild-type *atgl-1* RNAi vs *flp-7^tg^ atgl-1* RNAi | 0.504 | -5.7 to 2.9 |
| 1H | *flp-7^tg^* vector RNAi vs *flp-7^tg^ atgl-1* RNAi | p<0.001 | 3.5 to 7.6 |
| 1I | wild-type vector RNAi vs *flp-7^tg^* vector RNAi | 0.009 | 1.92 to 11.8 |
| 1I | wild-type *atgl-1* RNAi vs *flp-7^tg^ atgl-1* RNAi | 0.302 | -5.8 to 1.9 |
| 1I | *flp-7^tg^* vector RNAi vs *flp-7^tg^ atgl-1* RNAi | p<0.001 | 4.4 to 13.2 |
| 1J | wild-type vs *flp-7^tg^* | 0.6421 | -0.5 to 1.0 |
| 1K | fasted wild-type vs fasted *flp-7^tg^* | 0.9626 | -1.7 to 2.1 |
| 1K | re-fed wild-type vs Re-fed *flp-7^tg^* | 0.9870 | -1.8 to 2.0 |
| 1L | wild-type vs *flp-7^tg^* | 0.6881 | -0.3 to 0.8 |
| 2A | wild-type vs *flp-7^tg^* | 0.0825 | 0.4 to 1.8* |
| 2D | wild-type vector RNAi vs *flp-7^tg^* vector RNAi | p<0.001 | 86.0 to 155.7 |
| 2D | wild-type *atgl-1* RNAi vs *flp-7^tg^ atgl-1* RNAi | 0.0044 | 11.2 to 80.9 |
| 2D | *flp-7^tg^* vector RNAi vs *flp-7^tg^ atgl-1* RNAi | p<0.001 | -117.1 to -47.5 |
| 2E | wild-type vector RNAi vs *flp-7^tg^* vector RNAi | 0.0258 | 0.1 to 1.5 |
| 2E | wild-type *atgl-1* RNAi vs *flp-7^tg^ atgl-1* RNAi | 0.6506 | -0.4 to 0.6 |
| 2E | *flp-7^tg^* vector RNAi vs *flp-7^tg^ atgl-1* RNAi | 0.0029 | 0.5 to 1.5 |
| 2E | wild-type vector RNA vs wild-type *atgl-1* RNAi | 0.0491 | -0.8 to -0.01 |
| 2F | wild-type vector RNAi vs *flp-7^tg^* vector RNAi | p<0.001 | 96.2 to 145.6 |
| 2F | *flp-7^tg^* vector RNAi vs *flp-7^tg^ atfs-1* RNAi | p<0.001 | -164.1 to -114.7 |
| 2F | *flp-7^tg^* vector RNAi vs *flp-7^tg^;atfs-1 atgl-1* RNAi | p<0.001 | -175.8 to -126.5 |
| 2F | wild-type *atfs-1* RNAi vs *flp-7^tg^ atfs-1* RNAi | 1.0000 | -25.6 to 23.8 |
| 2F | *atfs-1* *atgl-1* RNAi vs *flp-7^tg^;atfs-1 atgl-1* RNAi | 0.9507 | -32.2 to 17.1 |
| 2G | wild-type vector RNAi vs *flp-7^tg^* vector RNAi | 0.0258 | 0.1 to 1.5 |
| 2G | *flp-7^tg^* vector RNAi vs *flp-7^tg^ atfs-1* RNAi | 0.0030 | -1.6 to -0.4 |
| 2G | *flp-7^tg^* vector RNAi vs *flp-7^tg^;atfs-1 atgl-1* RNAi | 0.0014 | -1.7 to -0.5 |
| 2G | wild-type *atfs-1* RNAi vs *flp-7^tg^ atfs-1* RNAi | 0.9889 | -1.0 to 0.8 |
| 2G | *atfs-1* *atgl-1* RNAi vs *flp-7^tg^;atfs-1 atgl-1* RNAi | 0.9753 | -1.0 to 0.8 |
| S1 | vehicle vector RNAi vs 5-HT vector RNAi | 0.0455 | 0.01 to 0.6 |
| S1 | vehicle vector RNAi vs vehicle *atgl-1* RNAi | 0.0019 | -0.9 to -0.3 |
| S1 | 5-HT vector RNAi vs 5-HT *atgl-1* RNAi | 0.0002 | -1.2 to -0.6 |
| S1 | vehicle vector RNAi vs vehicle *atfs-1* RNAi | 0.0038 | -0.8 to -0.2 |
| S1 | 5-HT vector RNAi vs 5-HT *atfs-1* RNAi | p<0.001 | -1.1 to -0.6 |
| S1 | vehicle *atgl-1* RNAi vs 5-HT *atgl-1* RNAi | 0.7123 | -0.4 to 0.3 |
| S1 | vehicle *atfs-1* RNAi vs 5-HT *atfs-1* RNAi | 0.3579 | -0.3 to 0.1 |
| 3B | wild-type vector RNAi vs *flp-7^tg^* vector RNAi | p<0.001 | -83.2 to -57.0 |
| 3B | *atfs-1* vector RNAi vs *flp-7^tg^;atfs-1* vector RNAi | 0.9967 | -16.1 to 10.1 |
| 3B | wild-type vector RNAi vs wild-type *atgl-1* RNAi | p<0.001 | 17.2 to 43.4 |
| 3B | *flp-7^tg^* vector RNAi vs *flp-7^tg^ atgl-1* RNAi | p<0.001 | 7.0 to 93.2 |
| 3B | *atfs-1* *atgl-1* RNAi vs *flp-7^tg^;atfs-1 atgl-1* RNAi | 0.3357 | -7.2 to 45.8 |
| 3D | wild-type vector RNAi vs *flp-7^tg^* vector RNAi | p<0.001 | 19.6 to 56.0 |
| 3D | *flp-7^tg^* vector RNAi vs *flp-7^tg^ atfs-1* RNAi | p<0.001 | -65.1 to -28.8 |
| 3D | wild-type *atfs-1* RNAi vs *flp-7^tg^ atfs-1* RNAi | 0.6733 | -23.2 to 9.2 |
| 3E | wild-type vs *flp-7^tg^* | 0.0002 | 0.6 to 1.8 |
| 3E | *flp-7^tg^* vs *flp-7^tg^;atfs-1* | p<0.001 | -1.9 to -0.8 |
| 3E | *atfs-1* vs *flp-7^tg^;atfs-1* | 0.9489 | -0.7 to 0.5 |
| S2A | wild-type vehicle vs wild-type 5-HT | p<0.001 | -84.9 to -55.3 |
| S2A | wild-type 5-HT vs *atfs-1* 5-HT | p<0.001 | 11.8 to 41.4 |
| S2A | *atfs-1* vehicle vs *atfs-1* 5-HT | p<0.001 | -54.3 to -24.7 |
| S2B | wild-type vehicle vs wild-type 5-HT | 0.0341 | 0.1 to 1.3 |
| S2B | wild-type 5-HT vs *atfs-1* 5-HT | 0.0406 | 0.1 to 1.4 |
| S2B | *atfs-1* vehicle vs *atfs-1* 5-HT | 0.8302 | -0.5 to 0.6 |
| 4B | wild-type vs *flp-7^tg^* | p<0.001 | 20.2 to 55.5 |
| 4B | wild-type vs *hlh-11* | p<0.001 | 26.3 to 57.9 |
| 4B | *hlh-11* vs *hlh-11; flp-7^tg^* | 0.3587 | -25.8 to 5.8 |
| 4C | wild-type vs *flp-7^tg^* | 0.0083 | 0.3 to 2.1 |
| 4C | wild-type vs *hlh-11* | 0.0077 | 0.3 to 2.1 |
| 4C | *hlh-11* vs *hlh-11; flp-7^tg^* | 0.9159 | -1.1 to 0.7 |
| 4E | wild-type vector RNAi vs *flp-7^tg^* vector RNAi | p<0.001 | -82.4 to -57.8 |
| 4E | wild-type vector RNAi vs *hlh-11* vector RNAi | p<0.001 | -80.5 to -55.2 |
| 4E | *hlh-11* vector RNAi vs *hlh-11; flp-7^tg^* vector RNAi | 0.5673 | -20.4 to 4.9 |
| 4E | wild-type vector RNAi vs wild-type *atgl-1* RNAi | p<0.001 | 18.0 to 42.6 |
| 4E | *flp-7^tg^* vector RNAi vs *flp-7^tg^ atgl-1* RNAi | p<0.001 | 67.8 to 92.4 |
| 4E | *hlh-11* vector RNAi vs *hlh-11* *atgl-1* RNAi | p<0.001 | 65.6 to 90.9 |
| 4E | *flp-7^tg^;hlh-11* vector RNAi vs *flp-7^tg^;hlh-11* *atgl-1* RNAi | p<0.001 | 75.1 to 99.7 |
| 4E | *hlh-11* *atgl-1* RNAi vs *hlh-11; flp-7^tg^* *atgl-1* RNAi | 1.0000 | -10.9 to 13.7 |
| 4F | wild-type vector RNAi vs *hlh-11* vector RNAi | 0.037 | 0.2 to 6.4 |
| 4F | wild-type *atgl-1* RNAi vs *hlh-11 atgl-1* RNAi | 0.509 | -1.4 to 2.6 |
| 4F | *hlh-11* vector RNAi vs *hlh-11 atgl-1* RNAi | 0.002 | 1.9 to 7.6 |
| 4G | wild-type vector RNAi vs *hlh-11* vector RNAi | 0.009 | 1.9 to 11.8 |
| 4G | wild-type *atgl-1* RNAi vs *hlh-11 atgl-1* RNAi | 0.302 | -5.9 to 1.9 |
| 4G | *hlh-11* vector RNAi vs *hlh-11 atgl-1* RNAi | p<0.001 | 4.4 to 13.2 |
| 5C | wild-type vs *flp-7^tg^* | p<0.001 | -82.1 to -58.0 |
| 5C | wild-type vs HLH-11^ox^ | p<0.001 | 37.2 to 61.6 |
| 5C | *flp-7^tg^* vs *flp-7^tg^*;HLH-11^ox^ | 0.0078 | 95.0 to 119.1 |
| 5C | HLH-11^ox^ vs *flp-7^tg^*;HLH-11^ox^ | 0.1520 | -27.9 to 2.9 |
| 5D | wild-type vector RNAi vs HLH-11^ox^ vector RNAi | 0.0426 | -8.6 to -0.2 |
| 5D | HLH-11^ox^ vector RNAi vs *flp-7^tg^*;HLH-11^ox^ vector RNAi | 0.3660 | -2.8 to 7.2 |
| 5D | HLH-11^ox^ vector RNAi vs HLH-11^ox^ *atgl-1* RNAi | 0.8072 | -4.1 to 5.2 |
| 5E | wild-type vs HLH-11^ox^ | 0.0472 | -0.8 to -0.01 |
| 5E | wild-type vs *flp-7^tg^*;HLH-11^ox^ | 0.0379 | -0.8 to -0.03 |
| 5G | vehicle vs 5-HT set F1 and R1 | 0.0358 | 0.03 to 0.9 |
| 5G | vehicle vs 5-HT set F2 and R2 | 0.0027 | 0.3 to 1.2 |
| 5G | vehicle vs 5-HT set F3 and R3 | 0.0020 | 0.3 to 1.3 |
| 5I | wild-type vs Δ*cishlh-11* | p<0.001 | 103.8 to 135.0 |
| 6B | wild-type vs *flp-7^tg^* | 0.0042 | -96.8 to -19.2 |
| 6C | wild-type vector RNAi vs *flp-7^tg^* vector RNAi | 0.0170 | -0.7 to -0.1 |
| 6C | *flp-7^tg^* vector RNAi vs *flp-7^tg^;atfs-1* vector RNAi | 0.0280 | 0.1 and 0.2 |
| 6C | *atfs-1* vector RNAi vs *flp-7^tg^;atfs-1* vector RNAi | 0.1532 | 0.1 and 0.3 |
| 6C | *flp-7^tg^* vector RNAi vs *flp-7^tg^* *atgl-1* RNAi | 0.0413 | 0.01 to 0.3 |
| 7A | wild-type *atgl-1* RNAi vs *flp-7^tg^* *atgl-1* RNAi | 0.2278 | -0.5 to 0.2 |
| 7A | wild-type vector RNAi vs *flp-7^tg^* vector RNAi | 0.0258 | 0.1 to 1.5 |
| 7A | wild-type vector RNAi vs HLH-11^ox^ vector RNAi | 0.0462 | -1.0 to -0.01 |
| 7A | wild-type vector RNAi vs *flp-7^tg^*;HLH-11^ox^ vector RNAi | 0.0443 | -1.0 to -0.01 |
| 7A | wild-type vector RNAi vs *hlh-11* vector RNAi | 0.0007 | 0.5 to 1.7 |
| 7A | *hlh-11* vector RNAi vs *hlh-11;atfs-1* vector RNAi | 0.0009 | -2.0 to -0.6 |
| 7A | *flp-7^tg^*;*hlh-11* vector RNAi vs *flp-7^tg^*;*hlh-11;atfs-1* vector RNAi | 0.0136 | 0.2 to 2.0 |
| 7A | wild-type vector RNAi vs wild-type *atgl-1* RNAi | 0.0491 | -0.8 to -0.01 |
| 7A | *flp-7^tg^* vector RNAi vs *flp-7^tg^* *atgl-1* RNAi | 0.0029 | 0.5 to 1.5 |
| 7A | *hlh-11* vector RNAi vs *hlh-11* *atgl-1* RNAi | 0.0074 | -1.7 to -0.5 |
| 7A | *flp-7^tg^*;*hlh-11* vector RNAi vs *flp-7^tg^*;*hlh-11* *atgl-1* RNAi | 0.0001 | -1.9 to -0.7 |
| 7A | *hlh-11;atfs-1* vector RNAi vs *hlh-11;atfs-1* *atgl-1* RNAi | 0.9935 | -0.8 to 1.2 |
|  | *flp-7^tg^*;*hlh-11;atfs-1* vector RNAi vs *flp-7^tg^*;*hlh-11;atfs-1* *atgl-1* RNAi | 0.9974 | -1.2 to 0.9 |
| 7A | HLH-11^ox^ vector RNAi vs HLH-11^ox^ *atgl-1* RNAi | 1.000 | -0.3 to 0.3 |
| 7A | *flp-7^tg^*;HLH-11^ox^ vector RNAi vs *flp-7^tg^*;HLH-11^ox^ *atgl-1* RNAi | 0.9519 | -0.3 to 0.2 |
| 7C | wild-type vector RNAi vs *flp-7^tg^* vector RNAi | p<0.001 | 22.0 to 53.6 |
| 7C | *flp-7^tg^* vector RNAi vs *flp-7^tg^* *atfs-1* RNAi | p<0.001 | -62.6 to -31.2 |
| 7C | wild-type vector RNAi vs *hlh-11* vector RNAi | p<0.001 | 20.8 to 49.4 |
| 7C | wild-type vector RNAi vs *flp-7^tg^*;*hlh-11* vector RNAi | p<0.001 | 17.9 to 46.4 |
| 7C | *flp-7^tg^* *atfs-1* RNAi vs *flp-7^tg^*;*hlh-11 atfs-1* RNAi | p<0.001 | 23.9 to 51.9 |
| 7C | wild-type vector RNAi vs *hlh-11* *atfs-1* RNAi | p<0.001 | 9.4 to 44.1 |
| 7C | wild-type vector RNAi vs *flp-7^tg^*;*hlh-11 atfs-1* RNAi | p<0.001 | 14.6 to 42.9 |
| 7C | *hlh-11* *atfs-1* RNAi vs *flp-7^tg^*;*hlh-11 atfs-1* RNAi | p<0.001 | 13.9 to 64.8 |
| 7D | wild-type vs *flp-7^tg^* | 0.0122 | 0.3 to 2.1 |
| 7D | wild-type vs *hlh-11* | 0.0077 | 0.3 to 2.1 |
| 7D | wild-type vs *flp-7^tg^*;*hlh-11* | 0.0397 | 0.03 to 2.0 |
| 7D | *flp-7^tg^* vs *flp-7^tg^*;*atfs-1* | 0.0041 | -2.3 to -0.4 |
| 7D | *flp-7^tg^*;*atfs-1* vs *flp-7^tg^*;*hlh-11;atfs-1* | 0.0095 | 0.2 to 2.1 |
| 7D | wild-type vs *hlh-11;atfs-1* | 0.0431 | 0.02 to 1.4 |
| 7D | wild-type vs *flp-7^tg^*;*hlh-11;atfs-1* | 0.0285 | 0.1 to 2.00 |
| 7D | *hlh-11;atfs-1* vs *flp-7^tg^*;*hlh-11;atfs-1* | 0.5000 | -0.4 to 1.0 |
| 7D | *flp-7^tg^*;*hlh-11* vs *flp-7^tg^*;*hlh-11;atfs-1* | 0.8304 | -0.7 to 0.8 |
| 8B | *flp-7^tg^*;*atfs-1* vector RNAi vs *flp-7^tg^*;*hlh-11;atfs-1* vector RNAi | p<0.001 | -85.3 to -57.9 |
| 8B | *flp-7^tg^* vector RNAi vs *flp-7^tg^*;*hlh-11;atfs-1* vector RNAi | p<0.001 | -80.7 to -61.5 |
| 8B | *flp-7^tg^* vector RNAi vs *flp-7^tg^ atgl-1* RNAi | p<0.001 | 66.4 to 93.9 |
| 8B | *flp-7^tg^*;*hlh-11* vector RNAi vs *flp-7^tg^*;*hlh-11 atgl-1* RNAi | p<0.001 | 73.7to 101.1 |
| 8B | *flp-7^tg^;atfs-1* vector RNAi vs *flp-7^tg^;atfs-1 atgl-1* RNAi | p<0.001 | 29.3 to 56.7 |
| 8B | *flp-7^tg^*;*hlh-11;atfs-1* vector RNAi vs *flp-7^tg^*;*hlh-11;atfs-1 atgl-1* RNAi | p<0.001 | 77.8 to 105.3 |
| 8C | wild-type vs *flp-7^tg^*;*hlh-11;atfs-1* | p<0.001 | 0.5 to 1.9* |
| 8D | wild-type vs *hlh-11;atfs-1* | 0.1166 | 0.4 to 1.6* |
| 8E | wild-type vs *hlh-11* | 0.4056 | 0.2 to 1.7* |
| S3B | wild-type vector RNAi vs *hlh-11* vector RNAi | p<0.001 | -83.4 to -52.3 |
| S3B | *hlh-11* vector RNAi vs *hlh-11;atfs-1* vector RNAi | p<0.001 | 26.8 to 57.9 |
| S3B | *atfs-1* vector RNAi vs *hlh-11;atfs-1* vector RNAi | 0.0010 | -37.1 to -5.9 |
| S3B | wild-type vector RNAi vs wild-type *atgl-1* RNAi | p<0.001 | 14.7 to 45.8 |
| S3B | *hlh-11* vector RNAi vs *hlh-11* *atgl-1* RNAi | p<0.001 | 62.7 to 93.8 |
| S3B | *atfs-1* vector RNAi vs *atfs-1* *atgl-1*RNAi | p<0.001 | 3.3 to 34.4 |
| S3B | *hlh-11;atfs-1* vector RNAi vs *hlh-11;atfs-1* *atgl-1* RNAi | p<0.001 | 19.9 to 51.0 |

* indicates the 95% CI for the ratio of median survival
