# Supplementary material for: A feedback loop governs the relationship between lipid metabolism and longevity
Source: eLife. 2020 Oct 20;9:e58815. doi: 10.7554/eLife.58815 (PMC7575325; doi:10.7554/eLife.58815)
Supplement: Transparent reporting form [file elife-58815-transrepform.docx]

***eLife’s* transparent reporting form**

We encourage authors to provide detailed information *within their submission* to facilitate the interpretation and replication of experiments. Authors can upload supporting documentation to indicate the use of appropriate reporting guidelines for health-related research (see [EQUATOR Network](http://www.equator-network.org/%20)), life science research (see the [BioSharing Information Resource](https://biosharing.org/)), or the [ARRIVE guidelines](http://www.plosbiology.org/article/info:doi/10.1371/journal.pbio.1000412) for reporting work involving animal research. Where applicable, authors should refer to any relevant reporting standards documents in this form.

If you have any questions, please consult our Journal Policies and/or contact us: [editorial@elifesciences.org](mailto:editorial@elifesciences.org).

**Sample-size estimation**

- You should state whether an appropriate sample size was computed when the study was being designed
- You should state the statistical method of sample size computation and any required assumptions
- If no explicit power analysis was used, you should describe how you decided what sample (replicate) size (number) to use

Please outline where this information can be found within the submission (e.g., sections or figure legends), or explain why this information doesn’t apply to your submission:

Our manuscript utilizes several assays, each of which represents a readout at distinct levels of analysis. Therefore, each assay has been powered for sample size and statistical test based on the following: (i) pilot studies to assess the strength of the phenotype; (ii) minimum number of animals needed to detect significant differences (p<0.05). For each type of assay, we used the same number of animals for each genotype or condition so as not to over-power or under-power comparisons. This information has been stated in the Statistical Analyses sub-section within the Methods section. The sample size, statistical method and significance for each experiment is listed in the corresponding figure legend. All actual p-values are given in Supplemental File 1.

**Replicates**

- You should report how often each experiment was performed
- You should include a definition of biological versus technical replication
- The data obtained should be provided and sufficient information should be provided to indicate the number of independent biological and/or technical replicates
- If you encountered any outliers, you should describe how these were handled
- Criteria for exclusion/inclusion of data should be clearly stated
- High-throughput sequence data should be uploaded before submission, with a private link for reviewers provided (these are available from both GEO and ArrayExpress)

Please outline where this information can be found within the submission (e.g., sections or figure legends), or explain why this information doesn’t apply to your submission:

After the initial pilot or observational studies were conducted, each experiment was performed 2-4 times, and these were designated as biological replicates. The number of technical replicates depended on the type of experiment. For each type of experiment, the number of replicates and clarification between biological and technical replicates are stated within the relevant description in the Methods section. Wild-types were always included as controls for every experiment.

**Statistical reporting**

- Statistical analysis methods should be described and justified
- Raw data should be presented in figures whenever informative to do so (typically when N per group is less than 10)
- For each experiment, you should identify the statistical tests used, exact values of N, definitions of center, methods of multiple test correction, and dispersion and precision measures (e.g., mean, median, SD, SEM, confidence intervals; and, for the major substantive results, a measure of effect size (e.g., Pearson's r, Cohen's d)
- Report exact p-values wherever possible alongside the summary statistics and 95% confidence intervals. These should be reported for all key questions and not only when the p-value is less than 0.05.

Please outline where this information can be found within the submission (e.g., sections or figure legends), or explain why this information doesn’t apply to your submission:

All statistical analyses are stated in each figure legend and in each subsection within the Methods section. All N values, methods of multiple testing corrections are also detailed within the Figure Legends. Exact p-values are given in Supplemental File 1. The 95% confidence intervals of the differences between means are also stated in Supplemental File 1. We have reported raw data for all experiments in which it was informative to do so (for example, oxygen consumption rate, lifespan).

(For large datasets, or papers with a very large number of statistical tests, you may upload a single table file with tests, Ns, etc., with reference to sections in the manuscript.)

**Group allocation**

- Indicate how samples were allocated into experimental groups (in the case of clinical studies, please specify allocation to treatment method); if randomization was used, please also state if restricted randomization was applied
- Indicate if masking was used during group allocation, data collection and/or data analysis

Please outline where this information can be found within the submission (e.g., sections or figure legends), or explain why this information doesn’t apply to your submission:

Within a given genotype, samples were randomly assigned to experimental groups. For analyzing fluorescence readouts and fat content, *C. elegans* were blindly selected on a non-fluorescent dissecting microscope and transferred to slides to ensure no bias in sampling. All animals on the slide were imaged, with no exclusions. This information is given in the Methods section.

**Additional data files (“source data”)**

- We encourage you to upload relevant additional data files, such as numerical data that are represented as a graph in a figure, or as a summary table
- Where provided, these should be in the most useful format, and they can be uploaded as “Source data” files linked to a main figure or table
- Include model definition files including the full list of parameters used
- Include code used for data analysis (e.g., R, MatLab)
- Avoid stating that data files are “available upon request”

Please indicate the figures or tables for which source data files have been provided:

Our data analyses did not require complex transformations or second-order analyses. All genotypes and conditions were compared to wild-type, which was normalized to 100%, or given as an average raw value, and was always included in each experiment. Although we could tabulate all measurements, they would not convey more information than is presented in the graphs themselves, and would in fact be more cumbersome. Source data are actually already provided in every figure.
